# Effects of altered sialic acid biosynthesis on *N*-linked glycan branching and cell surface interactions

**DOI:** 10.1074/jbc.M116.764597

**Published:** 2017-04-19

**Authors:** Nam D. Pham, Poh-Choo Pang, Soumya Krishnamurthy, Amberlyn M. Wands, Paola Grassi, Anne Dell, Stuart M. Haslam, Jennifer J. Kohler

**Affiliations:** From the ‡ Department of Biochemistry, University of Texas Southwestern Medical Center, Dallas, Texas 75390-9038 and; the § Department of Life Sciences, Imperial College London, South Kensington Campus, London SW7 2AZ, United Kingdom

**Keywords:** aging, carbohydrate metabolism, galectin, N-linked glycosylation, sialic acid

## Abstract

GNE (UDP-GlcNAc 2-epimerase/ManNAc kinase) myopathy is a rare muscle disorder associated with aging and is related to sporadic inclusion body myositis, the most common acquired muscle disease of aging. Although the cause of sporadic inclusion body myositis is unknown, GNE myopathy is associated with mutations in GNE. GNE harbors two enzymatic activities required for biosynthesis of sialic acid in mammalian cells. Mutations to both GNE domains are linked to GNE myopathy. However, correlation between mutation-associated reductions in sialic acid production and disease severity is imperfect. To investigate other potential effects of GNE mutations, we compared sialic acid production in cell lines expressing wild type or mutant forms of GNE. Although we did not detect any differences attributable to disease-associated mutations, lectin binding and mass spectrometry analysis revealed that GNE deficiency is associated with unanticipated effects on the structure of cell-surface glycans. In addition to exhibiting low levels of sialylation, GNE-deficient cells produced distinct *N*-linked glycan structures with increased branching and extended poly-*N*-acetyllactosamine. GNE deficiency may affect levels of UDP-GlcNAc, a key metabolite in the nutrient-sensing hexosamine biosynthetic pathway, but this modest effect did not fully account for the change in *N*-linked glycan structure. Furthermore, GNE deficiency and glucose supplementation acted independently and additively to increase *N*-linked glycan branching. Notably, *N*-linked glycans produced by GNE-deficient cells displayed enhanced binding to galectin-1, indicating that changes in GNE activity can alter affinity of cell-surface glycoproteins for the galectin lattice. These findings suggest an unanticipated mechanism by which GNE activity might affect signaling through cell-surface receptors.

## Introduction

**Figure 1.**
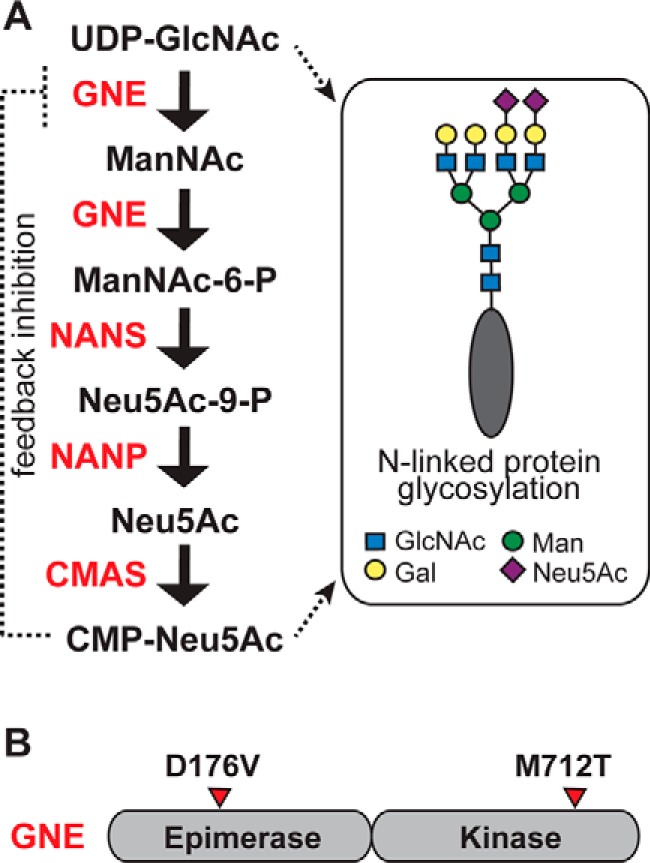
**GNE is a key regulator of sialic acid production.** *A*, GNE catalyzes two steps in sialic acid production: epimerization of UDP-GlcNAc, producing ManNAc, and phosphorylation of ManNAc, producing ManNAc-6-P. Subsequent steps convert ManNAc-6-P to Neu5Ac and then to CMP-Neu5Ac. Both UDP-GlcNAc and CMP-Neu5Ac are precursors to *N*-linked glycan biosynthesis. CMP-Neu5Ac inhibits the activity of GNE through a feedback mechanism. *B*, GNE is a bifunctional enzyme with an N-terminal epimerase domain and a C-terminal kinase domain. Mutations associated with GNE myopathy are found in both domains. The D176V and M712T mutations are examined here.

Sialic acid is an essential sugar in mammals; homozygous inactivation of the enzyme that catalyzes the first committed step in sialic acid biosynthesis, *Gne*,^3^ is embryonic lethal in mice (1). In humans, >100 mutations in *GNE* are linked to GNE myopathy, a rare disease of aging that is inherited in an autosomal recessive manner (2). Patients with GNE myopathy are normal at birth, but at ∼20 years of age they begin to develop relentlessly progressive asymmetric muscle wasting (2, 3). Despite clear association with *GNE* mutations, the mechanistic basis of GNE myopathy remains enigmatic. GNE is a bifunctional protein with an N-terminal epimerase domain that converts UDP-GlcNAc to *N*-acetylmannosamine (ManNAc) and a C-terminal kinase domain that phosphorylates ManNAc to ManNAc-6-P (Fig. 1*A*). Subsequent enzymatic transformations convert ManNAc-6-P to N-acetylneuraminic acid (Neu5Ac), the most abundant form of sialic acid in humans. Neu5Ac is further activated to the nucleotide sugar CMP-Neu5Ac, which regulates the activity of GNE through feedback inhibition. Both the GNE substrate, UDP-GlcNAc, and product, Neu5Ac, contribute to the biosynthesis of *N*-linked glycans (Fig. 1*A*). Mutations associated with GNE myopathy have been found in both the epimerase and kinase domains of GNE (Fig. 1*B*), but many only subtly alter enzyme activity *in vitro*; furthermore, reductions in enzyme activity do not correlate with disease severity (2–6).

Because of the key role of GNE as a regulator of sialic acid production, much work on GNE myopathy focused on the potential for changes in sialylation of cell surface glycoproteins and glycolipids (7–10). Although hyposialylation has been detected, the correlation between disease severity and reduction in cell surface sialylation is imperfect. Among humans who have GNE myopathy, many display sialic acid levels indistinguishable from unaffected humans (5). Similarly poor correlations between sialic acid levels and symptom severity can be observed in a mouse model based on the disease-associated D176V mutation to GNE (11). These mice are normal at birth but develop muscle weakness as they age. Remarkably, the muscle weakness can be prevented by providing sialic acid or its precursor ManNAc (12). Although treatment relieves symptoms, sialic acid levels in the mutant mice remain lower than those in control animals. Considering all of these observations, the incomplete correlation between sialic acid levels and disease severity suggests that mutations to GNE may have effects beyond hyposialylation.

In addition to its role in producing sialic acid, GNE is also directly linked to hexosamine metabolism. UDP-GlcNAc, the substrate of the GNE epimerase domain, is a product of the hexosamine biosynthetic pathway (HBP). UDP-GlcNAc levels and flux through the HBP control the extent of *N*-linked glycan branching (13). Thus, GNE activity has the potential to affect *N*-glycan structure by altering both sialic acid biosynthesis and flux through HBP.

Alterations in *N*-linked glycan branching have also been associated with age-related muscle weakness; homozygous inactivation of *Mgat5* in mice abolishes production of tetra-antennary *N*-linked glycans (13) and results in fewer muscle satellite cells and accelerated muscle weakness as the mice age (14). Taken together, existing data suggested the possibility that effects on HBP metabolism or on *N*-linked glycan structure could underlie at least some aspects of GNE myopathy pathogenesis. Notably, a study of patients with primary immunodeficiencies identified a genetic defect in the HBP that was associated with changes in *N*-linked glycan structure (15). These findings led us to examine whether GNE activity and GNE myopathy-associated mutations affect cell surface signaling through *N*-linked glycoproteins.

Here we use BJAB K20 cells that do not express GNE to examine the effects of GNE myopathy-associated mutations on *N*-linked glycan structure and function. We report that GNE deficiency, in addition to causing hyposialylation of *N*-linked glycans, also yields unanticipated effects on glycan structure, resulting in more branching and more polyLacNAc extension. Although GNE deficiency may affect UDP-GlcNAc levels, this effect is modest and seems unlikely to fully account for the observed changes in *N*-linked glycan structure. Indeed, GNE-independent sialic acid biosynthesis, induced by supplementation with ManNAc, can also yield reduced *N*-linked glycan branching. GNE-dependent effects on the *N*-linked glycan structure have functional impacts on interactions between cell surface proteins and the galectin lattice. However, these results do not directly explain the physiological effects of GNE-myopathy-associated mutations; elimination of GNE has a dramatic effect on *N*-linked glycan biosynthesis, but cells expressing wild-type GNE or a form of GNE with a myopathy-associated mutation produce similar *N*-linked glycans.

## Results

### Cell lines expressing mutant forms of GNE

We sought to assess the effects of GNE activity in a defined cell-based system. Because GNE myopathy is an autosomal recessive disease, we selected a cell line that lacks endogenous GNE activity. GNE is epigenetically silenced in BJAB K20 cells (16, 17). We introduced expression of wild-type GNE or a mutant form of GNE, GNE D176V (11), which harbors a GNE myopathy-associated mutation to the epimerase domain, or GNE M712T, which harbors a GNE myopathy-associated mutation to the kinase domain (18). We also prepared K20 cells expressing GNE kinase, an artificial gene lacking the epimerase domain (Fig. 1*B*). Stable expression of GNE, GNE D176V, GNE M712T, or GNE kinase in BJAB K20 cells was achieved by lentiviral infection. GNE-expressing BJAB K20 cells were also compared with parental BJAB K20 cells and to BJAB K88 cells, a related BJAB cell line that expresses active GNE (16). Cells were cultured in serum-containing media for all experiments, providing a potential exogenous source of sialic acid.

### Cells expressing wild-type GNE or mutant GNE produced sialic acid

**Figure 2.**
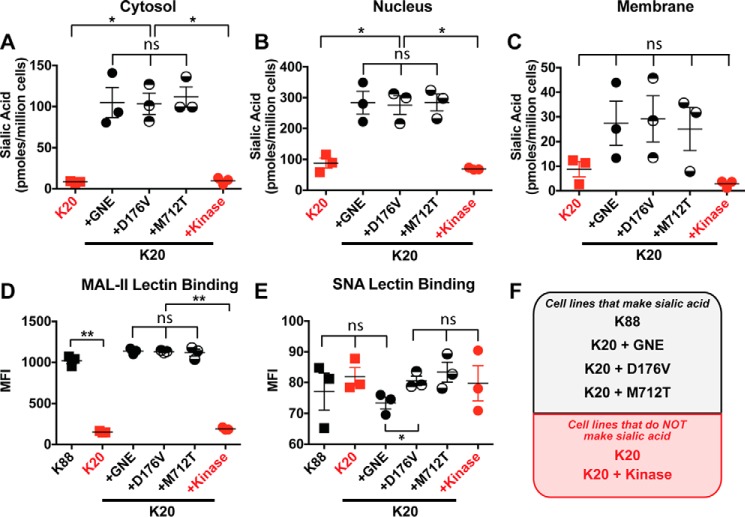
**Cells expressing GNE or GNE mutants produced sialic acid.** Cytosolic (*A*), nuclear (*B*), and membrane-associated (*C*) sialic acid were quantified by DMB derivatization with detection by fluorescent HPLC. Cell surface sialic acid was measured by flow cytometry using MAL-II lectin to detect α2–3-linked sialic acid (*D*) and SNA lectin to detect α2–6-linked sialic acid (*E*). *F*, cell lines labeled in *black* produce sialic acid, whereas cell lines labeled in *red* did not express an active GNE epimerase domain and consequently cannot synthesize sialic acid. For all panels, cells were cultured for 24 h, and data shown represent three biological replicates, with *error bars* depicting the mean and S.E. Each data point represents the MFI of a single sample, typically of 10,000 cells. Flow cytometry experiments were performed at least twice. Statistical significance determined by unpaired Welch's test: ** indicates a *p* value < 0.01, and * indicates a *p* value < 0.05. *ns* indicates difference not statistically significant.

First, we quantified sialic acid in the intracellular compartments of the cells. We used subcellular fractionation to isolate the nuclear and cytoplasmic fractions. Total compartmental sialic acid was quantified by 1,2-diamino-4,5-methylenedioxybenzene (DMB) derivatization and fluorescent HPLC analysis (Fig. 2, *A* and *B*) (19, 20). As expected, BJAB K20 parental cells and cells expressing GNE kinase had low levels of intracellular sialic acid, reflecting a lack of UDP-GlcNAc 2-epimerase activity. BJAB K20 cells expressing wild-type GNE or either GNE myopathy-associated mutant (D179V or M712T) had similar, high levels of intracellular sialic acid despite the fact that both mutations are known to result in reduced enzymatic activity (4, 9).

We used DMB derivatization to measure the total membrane-associated sialic acid in each cell line. We found that BJAB K20 parental cells and GNE kinase-expressing cells have similar low levels of membrane sialic acid, whereas cell lines expressing wild-type GNE or either of the GNE point mutants have similar high levels of sialic acid (Fig. 2*C*). We also used lectins to measure cell surface sialic acid. α2–3-Linked sialic acid was measured using the MAL-II lectin (21). Again, levels of α2–3-linked sialic acid were low in BJAB K20 parental cells and GNE kinase-expressing cells, whereas cell lines expressing wild-type GNE or a GNE point mutant exhibited similar, high levels of α2–3-linked sialic acid (Fig. 2*D*). All cell lines exhibited low levels of binding to SNA lectin, reflecting low levels of α2–6-linked sialic acid (Fig. 2*E*). Thus, all cell lines expressing a full-length form of GNE produced sialic acid (Fig. 2*F*), and we detected no measurable differences in sialic acid levels that could be attributed to disease-causing mutations.

### GNE-catalyzed sialic acid production results in altered N-glycan structure

**Figure 3.**
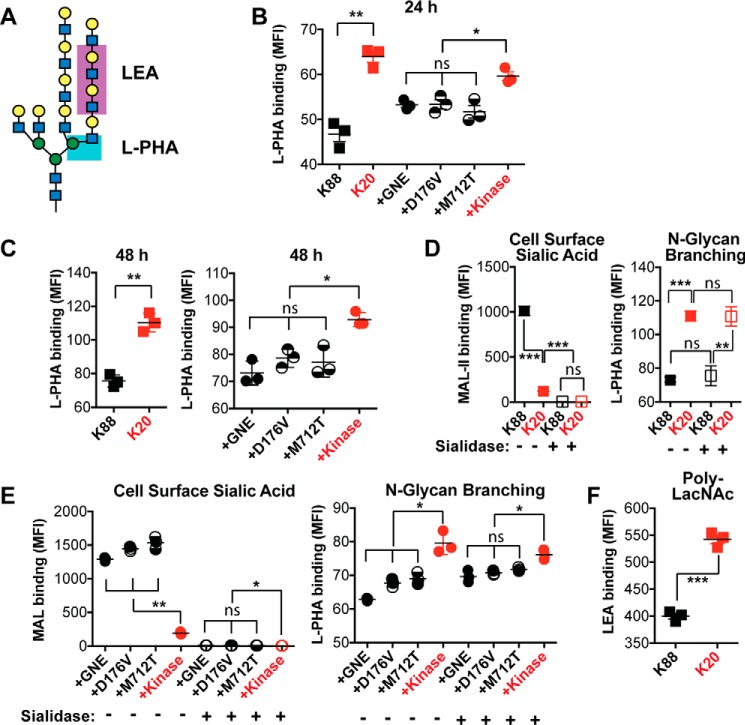
**Sialic acid production resulted in decreased *N*-linked glycan branching and decreased polyLacNAc extension.** *A*, L-PHA lectin recognizes the β1–6-GlcNAc branch of tri- and tetra-antennary *N*-linked glycans, whereas LEA lectin recognizes polyLacNAc structures. Cell surface L-PHA lectin binding was measured by flow cytometry after culturing cells for 24 (*B*) or 48 (*C*) h. To test whether sialylation affects L-PHA binding, cells were cultured for 24 h, then treated with *Arthrobacter ureafaciens* sialidase to remove sialic acid. Desialylation was confirmed by measuring MAL-II lectin binding by flow cytometry; L-PHA lectin binding to desialylated cells was also measured by flow cytometry. K20 and K88 cells are compared in *panel D*, whereas K20 cells expressing wild-type and mutant forms of GNE are compared in *panel E*. Cells were cultured for 24 h, then cell-surface LEA lectin binding was measured by flow cytometry (*F*). Cell lines labeled in *red* did not express an active GNE epimerase domain and did not synthesize sialic acid. Data shown represent three biological replicates with *error bars* depicting the mean and S.E. Each data point represents the MFI of a single sample, typically of 10,000 cells. Statistical significance determined by unpaired Welch's test: *** indicates a *p* value < 0.001, ** indicates a *p* value < 0.01, and * indicates a *p* value < 0.05. *ns* indicates difference not statistically significant.

The HBP supplies UDP-GlcNAc, the substrate for the UDP-GlcNAc 2-epimerase activity of GNE. In addition, the HBP also supplies UDP-GlcNAc for *N*-linked glycan biosynthesis. Indeed, the degree of branching of *N*-linked glycans is ultrasensitive to the intracellular concentration of UDP-GlcNAc (13). Relatively small increases in UDP-GlcNAc concentration can result in significant increases in the production of triantennary and tetra-antennary *N*-linked glycans. To test whether changes in GNE activity resulted in changes in *N*-linked glycan branching, we used the phytohemagglutinin-L lectin (L-PHA) to measure the levels of β1,6-GlcNAc-branched *N*-glycans (Fig. 3*A*). Flow cytometry analysis of L-PHA binding revealed less *N*-linked glycan branching in cell lines that produce sialic acid as compared with those that do not (Fig. 3, *B* and *C*). Differences in L-PHA binding were apparent after culturing the cells for either 1 or 2 days. To exclude the possibility that the presence or absence of sialic acid perturbed L-PHA binding, we also treated cells with sialidase before conducting the L-PHA-binding experiment. Treatment with sialidase resulted in effective desialylation but had no effect on L-PHA binding, indicating the sialylation status does not impact L-PHA binding (Fig. 3*D*). Furthermore, after desialylation, cell lines expressing the active forms of GNE still yielded lower levels of L-PHA binding than K20 cells that express only the GNE kinase domain and do not produce sialic acid (Fig. 3*E*). As in other experiments, the results for cell lines producing full-length wild-type GNE did not differ significantly from the results for cell lines expressing the disease-associated GNE mutants.

In addition to its role in *N*-linked glycan branching, UDP-GlcNAc is also required for polyLacNAc extension of *N*-linked glycan antennae. To investigate whether GNE activity affects polyLacNAc production, we measured binding of the *Lycopersicon esculentum* lectin (LEA) to BJAB K20 and BJAB K88 cells. We observed that GNE-expressing BJAB K88 cells exhibited less LEA binding than BJAB K20 cells, which lack GNE expression (Fig. 3*F*). Thus, differences in GNE activity were correlated with alterations in *N*-linked glycan structure, including increased *N*-linked glycan branching and increased polyLacNAc extension.

### Mass spectrometry analysis of N-linked glycans

Additional evidence for increases in *N*-linked glycan branching and polyLacNAc extension in cells lacking GNE activity was obtained by glycomics analyses using MALDI-TOF and MALDI-TOF/TOF mass spectrometry (22). A multiinstitutional study previously showed that MALDI-TOF is a reliable method for relative glycan quantification based on the signal intensities of permethylated glycans (23).

**Figure 4.**
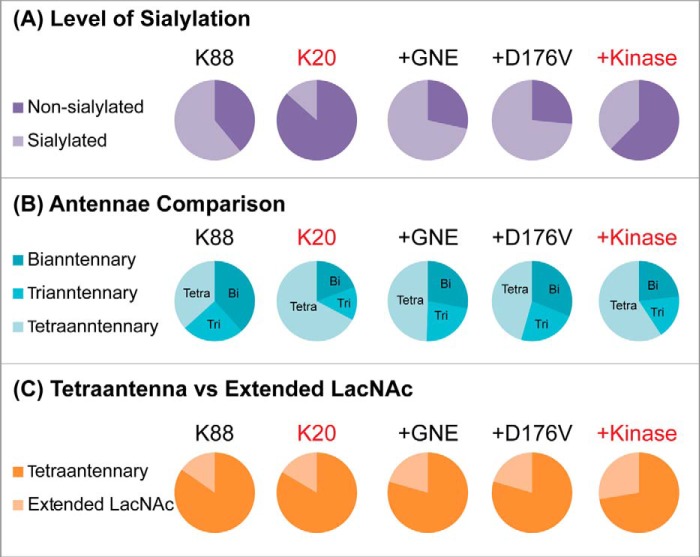
**Cells expressing active GNE produced more sialylated glycans and fewer tetra-antennary *N*-linked glycans.** Cells were cultured for 24 h, and membrane proteins were isolated. Permethylated *N*-linked glycans from K88, K20, +GNE, +D176V, and +Kinase cells were analyzed by MALDI-TOF and MALDI-TOF/TOF. Derived structural assignments, based on composition, tandem MS, and biosynthetic knowledge and relative abundances were used to assess complex *N*-glycan levels of sialylation (*A*), numbers of antennae (*B*), and tetra-antennary and extended LacNAc (*C*). A full list of structural compositions is available in supplemental Fig. 1 and Table 1. Mass spectrometry data are based on analysis of a single set of samples.

The MALDI-TOF glycomic profiles of BJAB K20 and BJAB K88 cells as well as BJAB K20 cells expressing wild-type GNE, GNE D176V, and GNE kinase are displayed in supplemental Fig. 1 together with glycan assignments and abundances (supplemental Table 1). Semiquantitative comparisons of these data confirm the loss of sialylation in the cells lacking GNE activity (Fig. 4*A*). Significantly, we observed higher levels of tetra-antennary glycans in cells lacking GNE activity (Fig. 4*B*), in agreement with the L-PHA-binding experiments described above. We also quantified the ratio of tetra-antennary *versus* extended LacNAc structures. Although this ratio did not differ dramatically among the cell lines (Fig. 4*C*), high mass extended LacNAc structures were more readily detected in GNE-deficient cells (supplemental Fig. 1 and Table 1), consistent with the LEA-binding data described above.

### GNE-dependent differences in N-linked glycan branching persist under low glucose conditions

**Figure 5.**
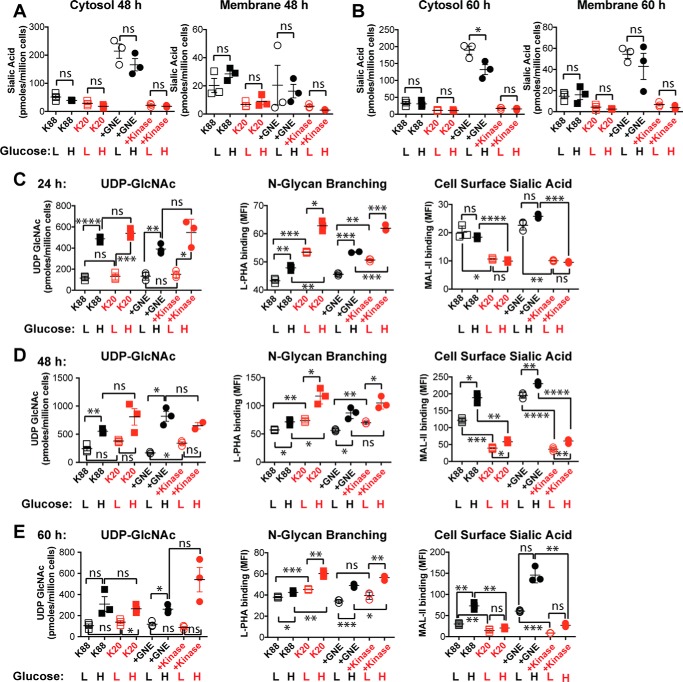
**Reduced glucose supplementation led to reduced UDP-GlcNAc levels and reduced *N*-linked glycan branching.** Cell lines were cultured with 0.5 g/liter glucose (low (*L*)) or 2 g/liter glucose (high (*H*)). Cytosolic and membrane-associated sialic acid were quantified by DMB derivatization with detection by fluorescent HPLC after cell culture in high and low glucose media for 48 h (*A*) and 60 h (*B*). In addition, UDP-GlcNAc levels were quantified by HPAEC with UV detection, and cell surface L-PHA and MAL-II lectin binding were measured by flow cytometry after culturing cells for 24 h (*C*), 48 h (*D*), or 60 h (*E*). Cell lines labeled in *black* produced sialic acid, whereas cell lines labeled in *red* did not express an active GNE epimerase domain and consequently could not synthesize sialic acid. For all panels, data shown represent three biological replicates, with *error bars* depicting the mean and S.E. For flow cytometry experiments, each data point represents the MFI of a single sample, typically of 10,000 cells. Flow cytometry experiments were performed at least twice. Statistical significance determined by unpaired Welch's test: **** indicates a *p* value < 0.0001, *** indicates a *p* value < 0.001, ** indicates a *p* value < 0.01, and * indicates a *p* value < 0.05. *ns* indicates difference not statistically significant.

We next sought to gain further insight into the relationship between the HBP and GNE-dependent effects on *N*-linked glycan branching. Because glucose supplies the HBP and ultimately sialic acid production, we compared BJAB cell lines that were cultured in “high” (2 g/liter) or “low” (0.5 g/liter) glucose-containing media (note that the high glucose condition reflects typical cell culture media and is used for all other experiments presented here, whereas the low glucose condition more accurately mimics a physiological glucose level). First, we evaluated whether glucose supplementation affected cytosolic or membrane sialic acid levels after culturing cells for 48 h (Fig. 5*A*) or 60 h (Fig. 5*B*). Levels of sialic acid were either unaffected or slightly reduced by the amount of glucose in the media, suggesting that flux through the HBP did not limit sialic acid production under these conditions. Next, we measured how glucose supplementation affected UDP-GlcNAc levels, *N*-linked glycan branching, and MAL-II binding after culturing cells for 24 h (Fig. 5*C*), 48 h (Fig. 5*D*), or 60 h (Fig. 5*E*). As expected, glucose supplementation resulted in dramatically increased UDP-GlcNAc levels, with the largest effects (about 5-fold increase) observed at early time points. Consistent with the higher UDP-GlcNAc levels, cells cultured in high glucose media demonstrated more L-PHA binding, reflecting more *N*-linked glycan branching. This effect was additive with the effect of active GNE expression. Thus, cells lacking GNE activity and cultured in high glucose media displayed the highest level of *N*-linked glycan branching, whereas cells expressing active GNE and cultured in low glucose media exhibited the lowest level of *N*-linked glycan branching. Effects on *N*-linked branching were less dramatic at later time points (Fig. 5*E*). Although glucose supplementation did not substantially affect total membrane sialic acid, as measured by DMB derivatization (Fig. 5, *A* and *B*), high glucose did lead to increased MAL-II binding at longer cell culturing times (Fig. 5, *D* and *E*), indicating that cell surface glycan structures are changed in a way that affects MAL-II binding.

### GNE-dependent differences in N-linked glycan branching persist during GlcNAc supplementation

**Figure 6.**
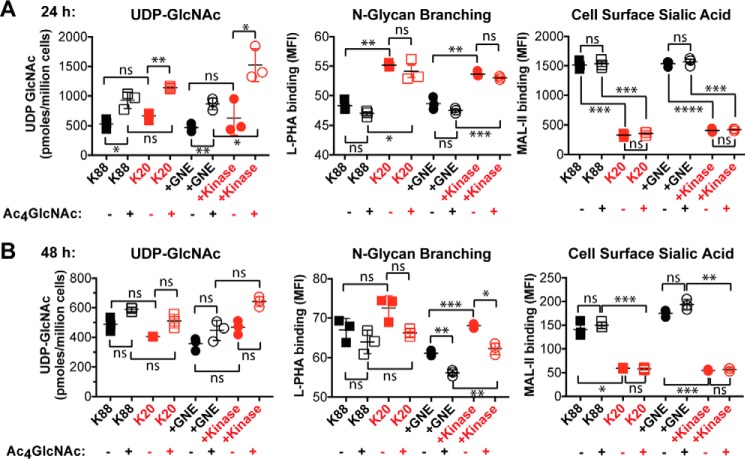
**Ac_4_GlcNAc supplementation can increase UDP-GlcNAc levels but did not increase *N*-linked glycan branching.** UDP-GlcNAc levels were quantified by HPAEC with UV detection, and cell surface L-PHA and MAL-II lectin binding was measured by flow cytometry after culturing cells for 24 h (*A*) or 48 h (*B*). Cell lines labeled in *black* produced sialic acid, whereas cell lines labeled in *red* did not express an active GNE epimerase domain and consequently could not synthesize sialic acid. For all panels, data shown represent three biological replicates, with *error bars* depicting the mean and S.E. For flow cytometry experiments, each data point represents the MFI of a single sample, typically of 10,000 cells. Flow cytometry experiments were performed at least twice. Statistical significance determined by unpaired Welch's test: **** indicates a *p* value < 0.0001, *** indicates a *p* value < 0.001, ** indicates a *p* value < 0.01, * indicates a *p* value < 0.05. *ns* indicates difference not statistically significant.

**Figure 7.**
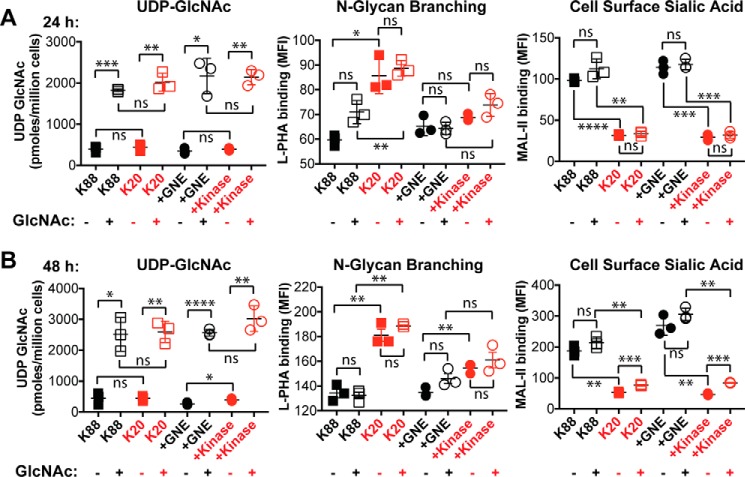
**Free GlcNAc supplementation dramatically increased UDP-GlcNAc levels and also increased *N*-linked glycan branching.** UDP-GlcNAc levels were quantified by HPAEC with UV detection, and cell surface L-PHA and MAL-II lectin binding was measured by flow cytometry after culturing cells for 24 h (*A*) or 48 h (*B*). Cell lines labeled in *black* produce sialic acid, whereas cell lines labeled in *red* did not express an active GNE epimerase domain and consequently cannot synthesize sialic acid. For all panels, data shown represent three biological replicates, with *error bars* depicting the mean and S.E. For flow cytometry experiments, each data point represents the MFI of a single sample, typically of 10,000 cells. Flow cytometry experiments were performed at least twice. Statistical significance determined by unpaired Welch's test: **** indicates a *p* value < 0.0001, *** indicates a *p* value < 0.001, ** indicates a *p* value < 0.01, * indicates a *p* value < 0.05. *ns* indicates difference not statistically significant.

Next, we tested whether changes in *N*-linked glycan branching could also be induced by culturing cells with 100 μm Ac_4_GlcNAc for either 24 h (Fig. 6*A*) or 48 h (Fig. 6*B*). Our hypothesis was that supplementation of GNE-expressing cells with Ac_4_GlcNAc would be expected to prevent UDP-GlcNAc depletion and maintain a high level of *N*-linked glycan branching. Indeed, including Ac_4_GlcNAc in the culture media resulted in a modest (∼2-fold) increase in UDP-GlcNAc levels, but interestingly and irrespective of GNE expression, Ac_4_GlcNAc supplementation had no effect on either cell surface sialic acid levels or *N*-linked glycan branching. We considered the possibility that using the protected form of GlcNAc (Ac_4_GlcNAc) might result in unanticipated effects due to incomplete deprotection and/or release of the acetyl-protecting groups (24, 25). Thus, we also cultured cells with 40 mm GlcNAc for either 24 h (Fig. 7*A*) or 48 h (Fig. 7*B*). Inclusion of free GlcNAc in the culture media resulted in robust (>5-fold at 48 h) increases in UDP-GlcNAc levels in all cell lines. GlcNAc supplementation was also able to support slightly increased *N*-linked glycan branching, particularly at the longer time point, but the effects were small relative to the differences between GNE-expressing and non-expressing cells.

### GNE-catalyzed sialic acid production did not affect O-GlcNAc levels

**Figure 8.**
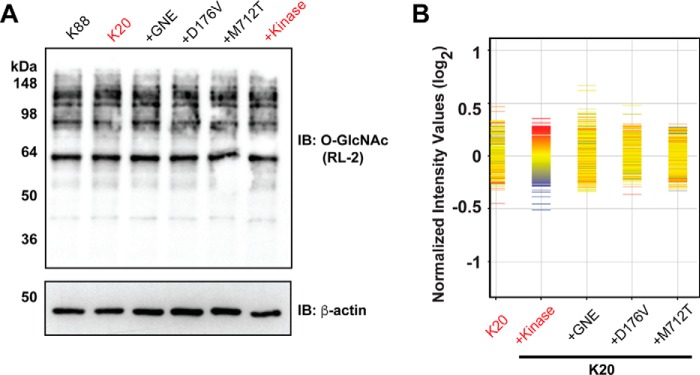
***O-*GlcNAc production and gene expression were not affected by GNE activity.** *A*, cell lines were cultured for 2 days. Lysates were probed by immunoblot (*IB*) using the *O-*GlcNAc-recognizing antibody, RL-2. *B*, profile plot of gene expression across different cell lines. Cell lines were cultured for 2 days. Duplicate samples of RNA were hybridized to an Illumina HumanHT-12 v4 BeadChip microarray. The normalized intensity values indicate log2 ratios of gene expression as compared with the average across all cell lines. Cells expressing GNE kinase were used as the reference cell line. *Red* indicates genes expressed *above baseline* in the reference cell line, whereas *blue* indicates genes expressed *below baseline* in the reference cell line. The *red* and *blue* colors are randomly distributed in the non-reference cell lines, indicating there are no gene expression changes that are correlated with GNE activity.

Although GNE expression did not dramatically affect UDP-GlcNAc levels (Fig. 5, *C–E*; 6, *A* and *B*; 7, *A* and *B*), we considered whether GNE expression was sufficient to affect a different biosynthetic process that depends on UDP-GlcNAc levels. Enzyme-catalyzed transfer of GlcNAc from UDP-GlcNAc to substrate proteins yields the *O-*linked β-*N*-acetylglucosamine (O-GlcNAc) post-translational modification. In this way nutrient flux through the HBP regulates protein *O-*GlcNAcylation (26). To test whether GNE activity affects protein *O-*GlcNAcylation, we prepared lysates from each cell line and analyzed them by immunoblot using an *O-*GlcNAc recognizing antibody, RL-2 (27). Consistent with the minor effect that GNE exerts on UDP-GlcNAc levels, we observed no dramatic differences in protein *O-*GlcNAcylation among the cell lines; however, small differences may be undetectable in this analysis (Fig. 8*A*).

### GNE-catalyzed sialic acid production exerted no overt effects on gene expression

Previous studies have reported that sialic acid metabolism leads to changes in gene expression (28, 29). To test whether changes in gene expression could account for the differences in *N*-linked glycan production that we observed, we isolated RNA from each cell line and analyzed gene expression by measuring hybridization to an Illumina microarray. Surprisingly, microarray analysis revealed that all cell lines had very similar levels of gene expression (Fig. 8*B*). Using the cells expressing GNE kinase as a reference, we detected no changes (>2-fold difference) in gene expression that correlated with sialic acid production. Under the conditions examined here, sialic acid production does not yield significant effects on gene expression in BJAB K20 cells.

### GNE-independent sialylation caused reduced N-linked glycan branching

**Figure 9.**
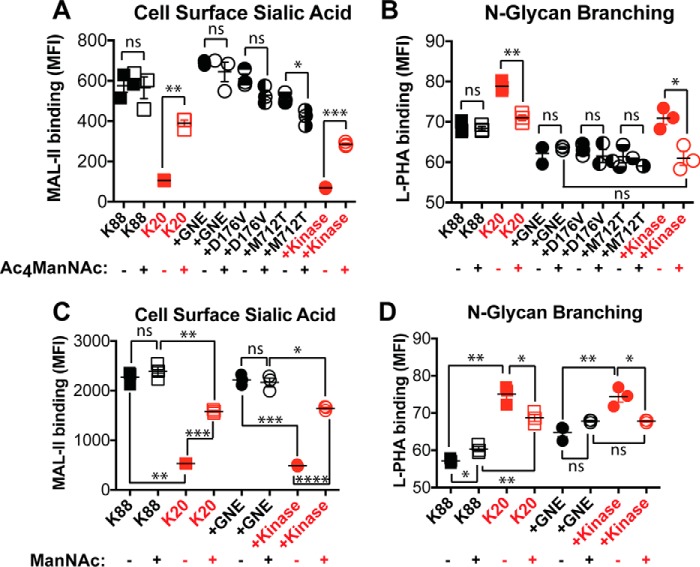
**Sialylation led to reduced *N*-linked glycan branching.** Cells were cultured with or without 100 μm Ac_4_ManNAc or 40 mm ManNAc for 24 h. The effect of Ac_4_ManNAc supplementation on sialylation and *N*-linked glycan branching was measured by flow cytometry detection of cell surface binding of the MAL-II lectin (*A*) and L-PHA lectin (*B*), respectively. The effect of ManNAc supplementation on sialylation and *N*-linked glycan branching was measured by flow cytometry detection of cell surface binding of the MAL-II lectin (*C*) and L-PHA lectin (*D*), respectively. Cell lines labeled in *red* did not express an active GNE epimerase domain and did not synthesize sialic acid. Data shown represent three biological replicates with *error bars* depicting the mean and S.E. Each data point represents the MFI of a single sample, typically of 10,000 cells. Flow cytometry experiments were performed at least twice. Statistical significance determined by unpaired Welch's test: **** indicates a *p* value < 0.0001, *** indicates a *p* value < 0.001, ** indicates a *p* value < 0.01, * indicates a *p* value < 0.05. *ns* indicates difference not statistically significant.

Because GNE expression had only a small effect on UDP-GlcNAc levels, we next investigated whether UDP-GlcNAc-independent pathways contribute to the mechanism by which GNE activity leads to reduced *N*-linked glycan branching. To do so, we intervened downstream of GNE, culturing cells with Ac_4_ManNAc, a compound that readily crosses the plasma membrane and is metabolized to sialic acid (30). Ac_4_ManNAc supplementation (100 μm) resulted in increased cell surface sialic acid in cell lines that lack GNE activity and had no effect on cell surface sialic acid in cell lines with active GNE (Fig. 9*A*). Ac_4_ManNAc supplementation had no effect on *N*-linked glycan branching in cell lines with active GNE but largely restored reduced L-PHA binding in cell lines that lack GNE activity (Fig. 9*B*). Similar results were observed when culturing cells with 40 mm ManNAc, which does not require intracellular deacetylation (Fig. 9, *C* and *D*). Thus, synthesis of sialic acid and sialylated glycans can cause reduced *N*-linked glycan branching even when UDP-GlcNAc is not being consumed by GNE.

### Increased GNE activity resulted in decreased galectin-1 binding

**Figure 10.**
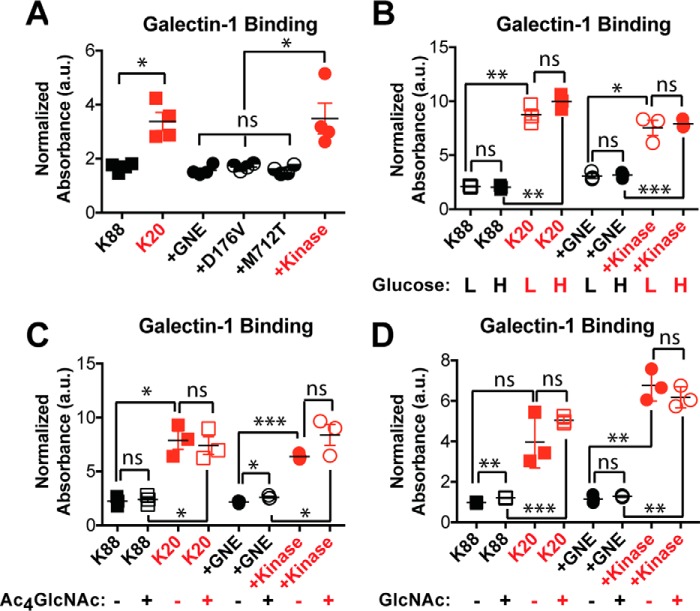
**GNE activity resulted in decreased galectin-1 binding.** Cells were cultured for 24 h in high glucose media (*A*), low or high glucose media (*B*), high glucose media supplemented with 100 μm Ac_4_GlcNAc (*C*), or high glucose media supplemented with free 40 mm GlcNAc (*D*). Binding of 3 μm biotinylated galectin-1 was measured using an ELISA assay and normalized to total protein content. Cell lines labeled in *red* did not express an active GNE epimerase domain and did not synthesize sialic acid. Data shown represent three or four biological replicates with *error bars* depicting the mean and S.E. Each experiment was performed at least twice. Statistical significance determined by unpaired Welch's test: *** indicates a *p* value < 0.001, ** indicates a *p* value < 0.01, * indicates a *p* value < 0.05. *ns* indicates difference not statistically significant.

We next investigated the functional consequences of GNE-induced changes in *N*-linked glycan branching. Alterations in *N*-linked glycan structure can have dramatic effects on membrane protein function. In particular, *N*-linked glycans are recognized by the extracellular galectin lattice, which controls surface retention and clustering of cell surface glycoproteins (31). Increases in *N*-linked glycan branching and polyLacNAc extension are associated with higher affinity for the galectin lattice (32). Thus, we tested whether GNE activity affected the ability of cell surface glycoproteins to engage galectin-1, a component of the galectin lattice. Cell lines expressing active GNE bound more poorly to galectin-1 (Fig. 10*A*), consistent with the decreased branching and reduced polyLacNAc extension that we observed in these cells. We did not detect any differences in galectin-1 binding that were attributable to the disease-causing GNE mutations. GNE-dependent differences in galectin-1 binding persisted in both high and low glucose conditions (Fig. 10*B*) and after supplementation with Ac_4_GlcNAc (Fig. 10*C*) or free GlcNAc (Fig. 10*D*).

## Discussion

**Figure 11.**
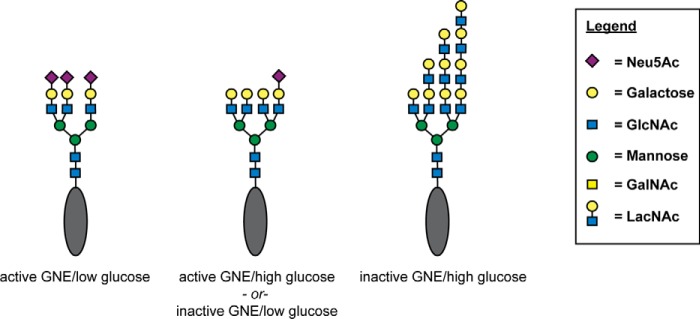
**GNE and glucose each alter *N*-linked glycan structure.** GNE deficiency causes decreased sialylation and high glucose results in higher levels of UDP-GlcNAc. These two changes additively affect *N*-linked glycan structure, resulting in less sialylation, more branching, and more polyLacNAc extension. Although all conditions result in heterogeneous mixtures of *N*-linked glycans, the schematics depict a representative glycan structure that might be expected to be abundant under the indicated set of conditions.

Here we assessed how GNE activity affects the structure of *N*-linked glycans. Lectin binding experiments and mass spectrometry analysis revealed three distinct effects. First, cells with GNE deficiency produced fewer sialylated *N*-linked glycans. Second, cells with GNE deficiency produced more highly branched *N*-linked glycans. Third, cells with GNE deficiency produced *N*-linked glycans with more polyLacNAc. *N*-Linked glycan branching and polyLacNAc extension can also be modulated by changes in UDP-GlcNAc levels (13), which are in turn regulated by levels of nutrient, specifically glucose, supplementation (33). Here we observed that glucose supplementation can work together with GNE deficiency in an additive manner to produce *N*-linked glycans with the highest level of branching. (Fig. 11).

We considered possible mechanisms by which GNE activity could result in changes in *N*-linked glycan structure. Initially, we focused on the possibility that GNE activity might deplete UDP-GlcNAc levels, resulting in less UDP-GlcNAc available to serve as substrates for enzymes, like MGAT5, which catalyzes production of branched glycans. Although K20 cells overexpressing full-length GNE typically displayed a lower steady-state UDP-GlcNAc level than cells overexpressing only the kinase domain (see Fig. 6*A*, for example), this effect was modest and not always significant. Furthermore, supplementing GNE-deficient cells with Ac_4_ManNAc or ManNAc was sufficient to cause a significant reduction in *N*-linked glycan branching, suggesting that the effects on *N*-linked glycan structure are not due solely to depletion of UDP-GlcNAc levels. Similarly, supplementing GNE-expressing cells with GlcNAc resulted in dramatic increases in UDP-GlcNAc levels but yielded no or small increases in *N*-linked glycan branching (Fig. 7). Indeed, it seems unlikely that the total amount of UDP-GlcNAc consumed by GNE would be sufficient to significantly perturb steady-state levels of this abundant metabolite. Thus, although GNE activity may affect UDP-GlcNAc levels and also flux through the HBP, these effects seem insufficient to mechanistically account for the entirety of observed changes in *N*-linked glycan structure. However, we cannot exclude the possibility that GNE activity perturbs local UDP-GlcNAc concentrations within the cell, which could limit the ability of key branching enzymes to access this substrate, a phenomenon observed by Mkhikian *et al.* (34).

The observation that GNE activity affects *N*-linked glycan branching adds to a growing body of literature identifying non-canonical roles for GNE (35–38). Some non-canonical roles appear to be independent of sialic acid synthesis (37) and may depend on the ability of GNE to engage in protein-protein interactions (36). In contrast, the effect on *N*-linked glycan branching appears to depend on sialic acid biosynthesis, as a similar effect can be induced by Ac_4_ManNAc or ManNAc supplementation in the absence of the GNE polypeptide. The increased polyLacNAc extension observed in GNE-deficient cells can reasonably be attributed to the absence of sialic acid that might otherwise cap glycans and prevent their extension, but the mechanism by which GNE activity (and sialic acid biosynthesis) affects *N*-linked glycan branching is less obvious. Indeed, the molecular basis for this effect remains to be elucidated. Possible explanations include reduced activity of MGAT5 toward prematurely sialylated substrates, free sialic acid itself acting as a signaling molecule, or sialylation affecting the activity of a glycoprotein, such as a Golgi-resident glycosyltransferase, involved in *N*-linked glycan maturation.

The degree of *N*-linked glycan branching regulates the ability of cell surface glycoproteins to interact with the galectin lattice, with more highly branched *N*-linked glycans engaging in more productive binding to galectins. Indeed, we found that cells expressing active GNE exhibited reduced binding by galectin-1, consistent with the decreased *N*-linked glycan branching. But surprisingly, glucose supplementation, which also substantially increased *N*-linked glycan branching (Fig. 5), did not affect galectin-1 binding under the conditions used here (Fig. 10). One possible explanation is that GNE deficiency and glucose supplementation each result in different types of branched structures, which cannot be distinguished by the crude analysis of L-PHA binding. Indeed, an important next step would be to perform additional mass spectrometry analysis to assess how glucose supplementation affects *N*-linked glycan structure. We also considered whether differences in sialylation could be responsible for the GNE-dependent differences in galectin-1 binding. Levels of α2,3-linked sialic acid varied among the examined cell lines, but galectin-1 binding is not sensitive to sialic acid in this linkage (39, 40). In contrast, galectin-1 binding is blocked by α2–6 sialylation (41, 42). Although α2–6 sialic acid levels were relatively low (Fig. 2*E*), differences in α2–6 sialylation could be responsible for GNE-dependent differences in galectin-1 binding. Differential expression of bisecting GlcNAc could also contribute to altered galectin-1 binding. Indeed, Chinese hamster ovary (CHO) cells with alterations in bisecting GlcNAc production exhibit changes in galectin binding (43). The mass spectrometry analysis we performed indicates that GNE expression results in increased levels of some glycans containing bisecting GlcNAc but decreased levels of others. Although no clear trend could be detected, alterations in bisecting GlcNAc expression could contribute to the altered galectin-1 binding that we observe. A final possibility is that the extended polyLacNAc structures that are observed in the absence of GNE activity could mediate the increased galectin-1 binding observed for GNE-deficient cells. Indeed, polyLacNAc is a preferred ligand for galectin-1 (42) and an important modulator of cell surface galectin interactions (34). Interactions with the galectin lattice are known to regulate cell surface retention of glycoproteins and thereby modulate signaling responses to extracellular cues (13, 32), suggesting the GNE activity could also play a role in regulating these processes. Although gene expression analysis did not reveal any baseline changes attributable to GNE expression, future efforts will test whether expression of GNE affects the ability of cells to respond to extracellular stimuli.

Interestingly, galectin-1 has been implicated in the development, repair, and regeneration of muscle tissue (44–48). Although initial examination of the galectin-1 null mouse revealed no muscle abnormalities, more recent studies indicate that myoblasts from the galectin-1 null mice have impaired fusion *in vitro* and reduced *in vivo* fiber diameter and myonuclear number when compared with wild-type muscle (46, 49). Although the mechanism by which galectin-1 regulates muscle phenotypes is not well understood, these findings raise the possibility that the muscle abnormalities observed in GNE myopathies could result from dysregulation of galectin-1 binding in the setting of abnormal *N*-linked glycan branching.

The experiments reported here were initiated with the goal of exploring how mutations to GNE lead to GNE myopathies. Somewhat disappointingly, none of our analyses revealed significant differences between the cell line expressing wild-type GNE and cell lines expressing disease-causing mutant forms of GNE. However, these experiments did uncover a novel function for GNE; that is, controlling *N*-linked glycan branching. The results presented here suggest that in any case where aberrant sialylation is observed, it would be prudent to also examine the extent of *N*-linked glycan branching, as these changes may be correlated and accompanying phenotypic changes could result from either of the glycan structural changes. Indeed, our results are interesting in light of a recent study reporting a homeostatic mechanism in which branching and LacNAc extension are reciprocally regulated to maintain bioequivalent glycans (34). In our experiments three interrelated factors, nutrient (Glc) supplementation, GlcNAc levels, and GNE activity, could each be modulated to control the extent of *N*-linked glycan branching but with different functional consequences. Our results suggest that sialic acid production by GNE may impact the homeostatic glycan setpoint, which would be expected to affect signaling through *N*-glycans (50, 51). Additional work is needed to determine whether this previously unrecognized role of GNE in regulating *N*-linked glycan structure contributes to phenotypes observed in GNE myopathies.

## Experimental procedures

### Cell lines and culturing conditions

BJAB K20 and K88 cells (16) (obtained from Michael Pawlita (German Cancer Research Center) and James Paulson (The Scripps Research Institute)) were cultured in RPMI 1640 medium containing 2 mm glutamine, 2 g/liter d-glucose (Thermo Fisher 11875-093) and supplemented with 10% fetal calf serum, 100 units/ml penicillin, and 100 μg/ml streptomycin at 37 °C, 5% CO_2_ in a water-saturated environment. The above formulation is the high glucose condition and was used in most experiments unless otherwise stated. For low glucose media, cells were cultured in RPMI 1640 media containing 2 mm glutamine (Thermo Fisher 11879-020, 0 g/liter d-glucose) and supplemented with 500 mg/liter d-glucose (MP Biomedicals 199013), 10% fetal calf serum, 100 units/ml penicillin, and 100 μg/ml streptomycin. Ac_4_ManNAc was synthesized as previously described (52) and dissolved in ethanol as a 10 mm stock solution, and pure ethanol was used as a control. Ac_4_GlcNAc (TCI America A1459) was dissolved in DMSO as a 10 mm stock solution, and pure DMSO was used as a control. ManNAc (Sigma A8176) and GlcNAc were each dissolved in Dulbecco's phosphate-buffered saline (DPBS) and sterile-filtered as a 1 m stock solution, and pure DPBS was used as a control.

### Cloning of GNE and mutagenesis

*GNE* was cloned from human brain cDNA (Origene CH-1001) using the primers GNE forward (5′-AAAGCTAGCATGGAGAAGGGAAATAACC-3′) and GNE reverse (5′-TTTCTCGAGCTAGTAGATCCTGCGTGTTGTG-3′).

The resulting PCR product was cloned into pCR4 Blunt-TOPO vector (Invitrogen). Sequencing (UT Southwestern Sanger Sequencing Core) revealed that the sequence matched accession NM_005476.4. To prepare the gene for insertion into the lentiviral plasmid, restriction sites were added by performing PCR using the primers GNE-F-AgeI (5′-AAAACCGGTATGGAGAAGAATGGAAATAACC-3′) and GNE-R-SbfI (5′-TTTCCTGCAGGCTAGTAGATCCTGCGTGTTGTG-3′).

The PCR product was cloned into pCR4 Blunt-TOPO vector (Invitrogen). QuikChange mutagenesis (Agilent) was performed on this pCR4 Blunt-TOPO GNE plasmid with the following primers: GNE-D176V-F (5′-CATGTGTGAGGACCATGTTCGCATCCTTTTGGCAG-3′) and GNE-D176V-R (5′-CTGCCAAAAGGATGCGAACATGGTCCTCACACATG-3′); GNE-M712T-F (5′-CTGGGTGCTGCCAGCACGGTTCTGGACTAC-3′) and GNE-M712T-R (5′-GTAGTCCAGAACCGTGCTGGCAGCACCCAG-3′).

In addition, the kinase only construct was obtained using the primer AgeI-kinase-start (5′-AAAACCGGTATGACTCTAAGTGCCTTGGCCGTTG-3′) along with the GNE-reverse primer shown above. All plasmids were validated by sequencing and contained no secondary mutations. pCR4 Blunt-TOPO plasmids encoding wild-type GNE, GNE(D176), GNE(M712), and GNE kinase were digested with AgeI and SbfI. The resulting inserts were gel-purified and ligated into pRRL CAGpNFLAG BAF155 IRES GFP (Addgene, 24561), also digested with Age I and PstI. Restriction enzymes and ligase were purchased from New England BioLabs.

### Production of lentivirus and infection of K20 cells to express GNE constructs

Virus was produced using the third generation packaging system (53). Briefly, HEK-293T cells were transfected with a pRRL GNE IRES GFP plasmid (WT or mutants) accompanied with pRRE (12251, Addgene), pRSV-REV (12253, Addgene), and pMD2.G (C12259, Addgene) in the presence of FuGENE 6 (E2691, Promega) to generate lentivirus. Media were replaced with HI-BSA medium (12.8 g of BSA per liter of DMEM with 10% FBS and penicillin/streptomycin) after 20 h. After 2 days, supernatant containing lentivirus was harvested and filtered through a 0.45-μm polyvinylidene difluoride membrane, frozen on liquid nitrogen, and then stored at −80 °C. K20 cells (∼200,000) aliquots were incubated with 1:1, 1:5, and 1:10 lentivirus stock solution diluted with RPMI media and supplemented with 4 μg/ml Polybrene (AL-118, Sigma) to enhance infection efficiency. Cells were centrifuged at 500 × *g* for 2 h at 32 °C. Cells were resuspended in fresh media and placed in a 6-well tissue culture dish. After 48 h, successful infection was determined by detection of GFP fluorescence by flow cytometry. To achieve homogeneity, cells underwent two rounds of cell sorting with either an Aria or MoFlo cell sorter. Viral dilution that caused <20% of cells to be GFP-positive was used for the first round of sorting to increase the probability that cells were infected only once. After two rounds of sorting, all cell lines were >95% GFP-positive. GFP expression remained stable.

### Gene expression array

Cells were cultured for 2 days, then ∼1.6 million cells were harvested and washed twice with DPBS. RNA was extracted using the Aurum RNA extraction kit (Bio-Rad). For each cell line, 1 μg of RNA was submitted in duplicate to the UT Southwestern Microarray Core Facility. RNA quality was validated using an Agilent 2100 Bioanalyzer. RNA was labeled and amplified by using the Illumina TotalPrep RNA Amplication kit. Samples were then applied to an Illumina HumanHT-12 v4 BeadChip. Data were analyzed using BeadStudio and GeneSpring.

### Flow cytometry analysis

Lectin binding was evaluated by flow cytometry. The following reagents were used at the final concentrations indicated: SNA-biotin (Vector Laboratories B-1305, 20 μg/ml), MAL-II-biotin (Vector Laboratories B-1265, 10 μg/ml), allophycocyanin-streptavidin (APC-Strep) (Life Sciences, S-868), 5 μg/ml), LEA-biotin (Vector Laboratories B-1175, 1 μg/ml), L-PHA rhodamine (Vector Laboratories RL-1112, 20 μg/ml). K20 cells were cultured for the times indicated, with or without supplemental sugar, as indicated. Cells were harvested by centrifugation, washed twice with DPBS, then resuspended in DPBS at 2.0 × 10^6^ cells/ml. Then, 200 μl of cell suspension was transferred to V-bottom 96-well plate and pelleted by centrifugation. The cell pellets were incubated with 100 μl of lectin diluted in DPBS for 60 min at 4 °C (LEA incubation was 30 min) then washed with DPBS 3 times. When a secondary detection reagent was used, cells were incubated with 5 μg/ml allophycocyanin-streptavidin (Life Sciences, S-868) in DPBS for 45 min. Fluorescence was analyzed by flow cytometry on a FACSCalibur instrument (BD Biosciences) equipped with dual lasers at 488 nm and 635 nm. For sialidase experiments, before lectin staining, cells were incubated with Sialidase A (Prozyme GK80040) for 90 min at 37 °C in DPBS containing 0.1% BSA. Plots show the mean fluorescence intensity (MFI), typically for 10,000 cells.

### Cellular fractionation

Freshly harvested cells were fractionated as described (20). Briefly, cells were counted and harvested by centrifugation and then suspended in hypotonic lysis buffer containing protease inhibitors for 15 min on ice. Cells were lysed by extrusion through a 25-gauge needle. Nuclei and unbroken cells were removed from the post-nuclear supernatant by two rounds of centrifugation at 1000 × *g* for 15 min at 4 °C. Next, the post-nuclear supernatant was transferred to heavy-walled polycarbonate tubes and centrifuged at 100,000 × *g* for 1 h in a Beckman TLA 120.2 rotor. The supernatant was designated the cytosolic fraction. The pellet was washed twice with 400 μl cold hypotonic lysis buffer followed by centrifugation at 100,000 × *g* for 1 h after each wash. The remaining pellet was designated the membrane fraction. Samples were quickly frozen in liquid nitrogen, and the solvent was removed by vacuum overnight.

### Quantification of DMB-derivatized sialic acids

To release sialic acids, 2 m acetic acid was added to each of the membrane-bound (50 μl) and cytosolic samples (100 μl). Solutions were incubated at 80 °C for 2 h. Samples were cooled to room temperature, then DMB reaction solution (7.0 mm DMB, 1.4 m acetic acid, 0.75 m 2-mercaptoethanol, 18 mm Na_2_S_2_O_3_) was added to each sample (40 μl for membrane fraction and 80 μl for cytosolic fraction). After a 2-h incubation at 50 °C, 0.2 m NaOH was added to each sample (10 μl for membrane fraction and 20 μl for cytosolic fraction). Samples were filtered through 10-kDa molecular-weight-cutoff filters by centrifugation, and the resulting flow-through was stored at −20 °C in the dark until analysis. Generally, 2 μl of the derivatized material was diluted with 98 μl of double distilled H_2_O and analyzed by fluorescence HPLC. Quantification was performed relative to known standards as follows. Calibration curves were prepared by injecting between 50–750 fmol of DMB-Neu5Ac on a Dionex Acclaim® Polar Advantage C16 5-μm, 4.6 × 250-mm column attached to a Dionex Ultimate 3000 HPLC with a fluorescence detector. Separation was performed using a gradient of 2–90% acetonitrile (double distilled H_2_O) with fluorescence detection (excitation 373 nm, emission 448 nm). Linearity of the fluorescence signal of DMB-Neu5Ac was confirmed in the range of 50–750 fmol. Samples were analyzed in the same manner. Linear regression analysis using the DMB-Neu5Ac standard curves was used to calculate the amount of DMB-Neu5Ac present in experimental samples.

### Cell-based ELISA

Cells were cultured in media for the indicated amounts of time. Cells (400,000) in a volume of 200 μl were harvested and placed in a 96-well v-bottom plate. Cells were chilled on ice for 15 min and then washed 3 times with DPBS. Cells were then incubated with 3 μm biotinylated galectin-1 (54) (55) (a kind gift from Linda Baum, UCLA) in DPBS for 30 min on ice. Cells were pelleted by centrifugation, then washed with cold DPBS three times. Half of each sample (100 μl) was transferred to another 96-well v-bottom plate. The protein content of these cells was analyzed by BCA assay (Pierce), and the absorbance was measured at 562 nm. The remaining half of each sample (100 μl) was incubated with streptavidin-peroxidase in DPBS containing 1% BSA for 1 h at 4 °C. After washing 3 times with DPBS, cells incubated with 100 μl of *o*-phenylenediamine (OPD) solution (20 ml of 50 mm phosphate-citrate buffer, pH 5.0, 20 mg of OPD tablet (Sigma P5412)) and 20 μl of 30% H_2_O_2_. Reactions were quenched with 50 μl of 5 m H_2_SO_4_. Absorbance at 490 nm and 650 nm was measured using a Biotek Synergy H1 Hybrid Reader. Galectin-1 binding was normalized to cell content, calculated by the following formula: galectin-1 binding = (*A*_490_ − *A*_650_)/*A*_562_.

### Glycan mass spectrometry

Cells (40 million per sample) were pelleted by centrifugation and frozen in liquid nitrogen and stored at −80 °C. Membrane proteins were isolated as follows. Cell pellets were suspended in 2 ml of lysis buffer containing 50 mm Tris-HCl, pH 7.4, 0.1 m NaCl, 1 mm EDTA, and a protease inhibitor mixture (Roche Diagnostics). After 20 min of incubation on ice, samples were homogenized using a Polytron homogenizer (15 s, 7 times on ice bath). Homogenates were centrifuged at 2000 × *g* for 20 min at 4 °C to precipitate nuclei. The supernatant was diluted with 2 ml of Tris-buffer (50 mm Tris-HCl, pH 7.4, 0.1 m NaCl), then membranes were pelleted by ultracentrifugation at 120,000 × *g* for 80 min at 4 °C. The supernatant was discarded, and the membrane pellet was resuspended in 100 μl of Tris buffer. After adding 400 μl of Tris-buffer containing 1% (v/v) Triton X-114, the suspended mixture was homogenized by vigorous pipetting. The homogenate was chilled on ice for 10 min, then incubated at 37 °C for 20 min and, finally, centrifuged at 2000 × *g* for 2 min. The upper, aqueous phase was removed. The lower, detergent phase was mixed with 1 ml of ice-cold acetone and stored at −20 °C overnight to precipitate (glyco)proteins. After centrifugation at 1940 × *g* for 2 min, the precipitated cell membrane (glyco)proteins were stored at −20 °C before analysis.

*N*-Linked glycans were released and processed as described previously (22). Briefly, precipitated (glyco)proteins were reduced, carboxymethylated, and digested with trypsin. The digests were then purified by C18-Sep-Pak (Waters Corp., Hertfordshire, UK). *N*-Glycans were released by peptide *N*-glycosidase F (E.C. 3.5.1.52; Roche Applied Science) digestion. Released *N*-glycans were permethylated using the sodium hydroxide procedure and purified by C18-Sep-Pak. The permethylated *N*-glycans were then dissolved in methanol before an aliquot was mixed at a 1:1 ratio (v/v) with 10 mg/ml 3,4-diaminobenzophenone in 75% acetonitrile. The glycan-matrix mixture was spotted on a stainless steel target plate and dried in vacuum. MALDI-TOF MS and MALDI-TOF/TOF MS/MS data were obtained using a 4800 MALDI-TOF/TOF mass spectrometer (AB Sciex UK Limited) in the positive-ion mode. For MS/MS, the collision energy was set at 1 kV, and argon was used as the collision gas. The obtained MS and MS/MS data were viewed and processed using Data Explorer 4.9 (AB Sciex UK Ltd).

### Details of experimental data used to define glycan structures

All *N*-glycans were assumed to have a core of Manα1-6(Manα1–3)Manβ1–4GlcNAcβ1–4GlcNAc based on known biosynthetic pathways and susceptibility to peptide *N*-glycosidase F digestion. Monosaccharide compositions in terms of numbers of Hex, HexNAc, etc. derived from MALDI-MS in the positive-ion mode of molecular ions of peptide *N*-glycosidase F-released permethylated species were assigned manually. MALDI-TOF/TOF MS/MS fragmentation was from the following molecular ions: *m*/*z* 2938, 3387, 3748, 3838, 4197, and 4285. Fragment ions were identified manually and with the assistance of the Glycoworkbench tool (Version 1.2) (56). Details provided were guided by MIRAGE (57).

### Isolation of intracellular metabolites

Cells were cultured for various times as indicated, then pelleted by centrifugation and counted. Cells (typically 2–5 × 10^6^) were pelleted, washed with cold DPBS twice, and flash-frozen in liquid nitrogen. Cells were lysed with 80% “super-cold” methanol (on dry ice) (58). Lysates was centrifuged at 16,000 − *g* for 15 min at 4 °C. The supernatant was flash-frozen in liquid nitrogen and dried under vacuum for 3–5 h. If not analyzed immediately, the intracellular metabolites were stored at −80 °C before analysis.

### Analysis of intracellular metabolites by HPAEC

Metabolite pellets were resuspended in 40 mm sodium phosphate buffer (pH 7.4; 100 μl per sample) and filtered through an Amicon® Ultra centrifugal filter unit (Millipore, 10,000 molecular weight cutoff; 2 times at 14,000 × *g* for 15 min at 4 °C). Filtrates were analyzed by HPAEC (ICS-3000 system, Dionex) with CarboPac™PA1 column (Dionex) with a pulsed amperometry detector (PAD) and UV-detector in-line (59, 60). Typically, 20 μl of metabolite was injected into the sample loading loop before the sample entered a guard column (Dionex, 4 × 50 mm) and then an analytical column (Dionex, 4 × 250 mm). The eluents used were 1.0 mm NaOH (Buffer C) and 1.0 m NaOAc and 1.0 mm NaOH (Buffer D). Low-carbonate NaOH (50% in water) was obtained from Fisher (SS254-1), and NaOAc was from Sigma (71183). HPAEC was performed with a flow rate = 1 ml/min, and the following gradient elution was performed: *T*_0 min_ = 95% C, *T*_5 min_ = 85% C, *T*_15 min_ = 70% C, *T*_20 min_ = 60% *C*, T_45 min_ = 60% *C*, T_50 min_ = 0% *C*, T_60 min_ = 0% *C*, T_65 min_ = 95% *C*, and T_75 min_ = 95% *C*. UDP-GlcNAc standards (100 μm, 50 μm, 25 μm, 10 μm, 4 μm, and 2.5 μm) were analyzed on the same day as cellular samples. UDP-GlcNAc peak areas were calculated, and raw data were converted to pmol of UDP-GlcNAc by comparing to a standard curve generated by analyzing the peak areas of the UDP-GlcNAc standards. Data were normalized to cell number.

## Author contributions

N. D. P. and J. J. K. designed the study and wrote the paper. N. D. P. performed and analyzed the experiments shown in Figs. 2, 3, and 5–10. S. K. performed and analyzed the flow cytometry experiments shown in Figs. 5–7 and 9. A. M. W. performed and analyzed UDP-GlcNAc measurements and immunoblot experiments shown in Figs. 5–8. P.-C. P., P. G., S. M. H., and A. D performed and analyzed the experiments shown in Fig. 4 and supplemental Fig. 1 and Table 1. All authors reviewed the results and approved the final version of the manuscript.

## Supplementary Material

Supplemental Data

## Footnotes

3 The abbreviations used are:

GNE: UDP-GlcNAc 2-epimerase/ManNAc kinase
ManNAc: *N*-acetylmannosamine
ManNAc-6-P: ManNAc-6-phosphate
UDP-GlcNAc: uridine diphosphate *N*-acetylglucosamine
HBP: hexosamine biosynthetic pathway
polyLacNAc: poly-*N*-acetyllactosamine
Neu5Ac: *N*-acetylneuraminic acid
DMB: 1,2-diamino-4,5-methylenedioxybenzene
HPAEC: high performance anion exchange chromatography
SNA: *Sambucus nigra* agglutinin
MAL-II: *Maackia amurensis* lectin II
O-GlcNAc: *O-*linked β-*N*-acetylglucosamine
L-PHA: leukocyte phytohemagglutinin
LEA: *L. esculentum* agglutinin
MFI: mean fluorescence intensity.
