# Supplementary material for: Effects of altered sialic acid biosynthesis on N-linked glycan branching and cell surface interactions
Source: J Biol Chem. 2017 Apr 19;292(23):9637–51. doi: 10.1074/jbc.M116.764597 (PMC5465488; doi:10.1074/jbc.M116.764597)
Supplement: Supplemental Data [file supp_292_23_9637__index.html]

Effects of altered sialic acid biosynthesis on N-linked glycan branching and cell surface interactions — Effects of altered sialic acid biosynthesis on N-linked glycan branching and cell surface interactions — Sialic acid biosynthesis and N-glycan structure — Supplemental Data 

# Effects of altered sialic acid biosynthesis on *N*-linked glycan branching and cell surface interactions

## Supplemental Data

- Supplementary Figure 1, Supplementary Table 1 (.pdf, 4.1 MB) - MALDI-MS analysis of K88, K20, +GNE, +D176V and +Kinase cells.
