# Supplementary material for: Effects of altered sialic acid biosynthesis on N-linked glycan branching and cell surface interactions
Source: J Biol Chem. 2017 Apr 19;292(23):9637–51. doi: 10.1074/jbc.M116.764597 (PMC5465488; doi:10.1074/jbc.M116.764597)
Supplement: Supplemental Data [file 10.1074_M116.764597_jbc.M116.764597-1.pdf]

Supplementary for Sialic Acid Biosynthesis Impacts Hexosamine Pathway Metabolism and N-linked Glycan Structure

SUPPLEMENTARY FIGURE LEGENDS

Supplementary Figure 1. MALDI-MS analysis of K88, K20, +GNE, +D176V and +Kinase cells. Permethylated N-linked glycans from K88, K20, +GNE, +D176V and +Kinase cells were analyzed by MALDI-TOF mass spectrometry. All molecular ions are in form of $[M+Na]^+$.

(1-1a) K88

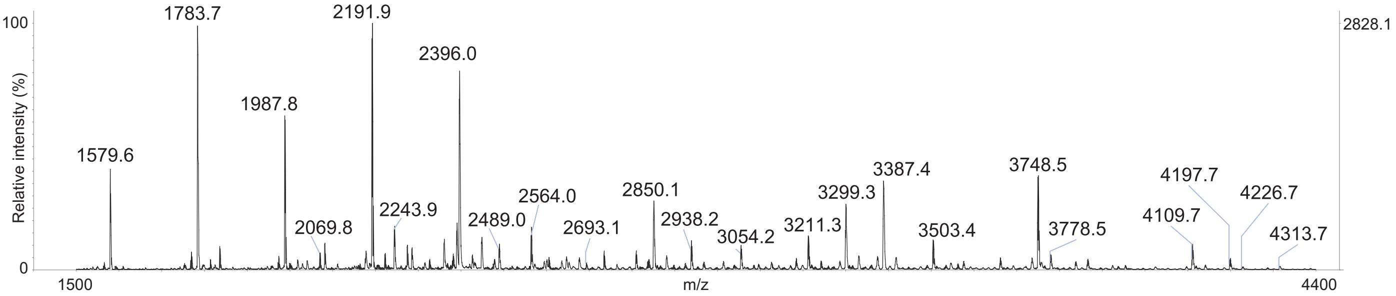

(1-2a) K20

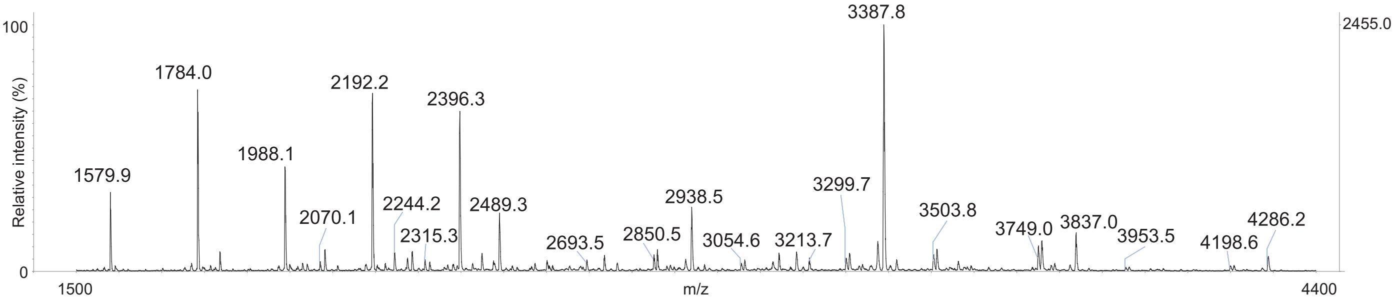

(1-3a) +GNE

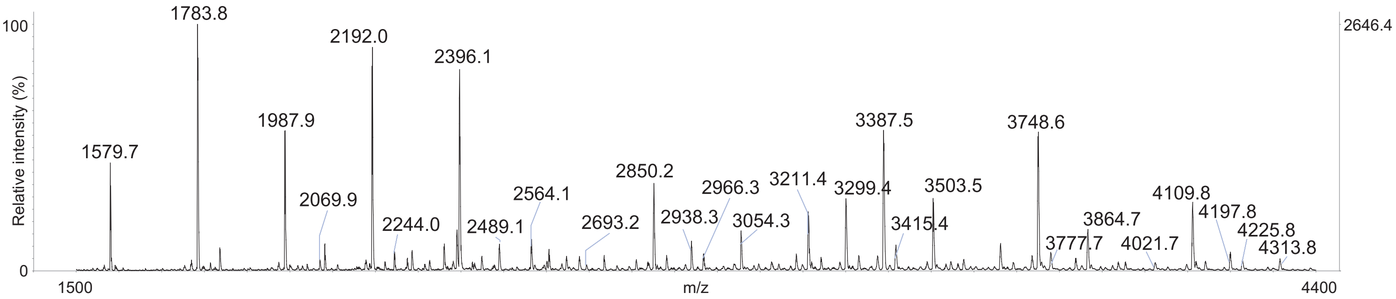

(1-4a) +D176V

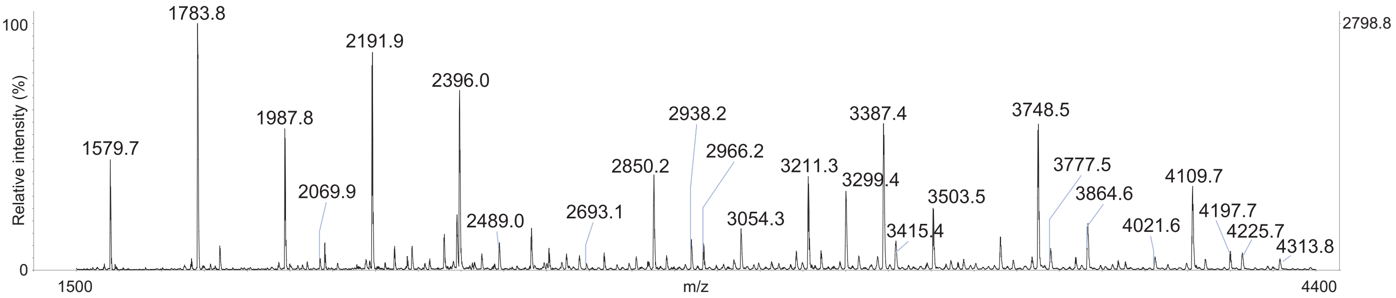

(1-5a) +Kinase

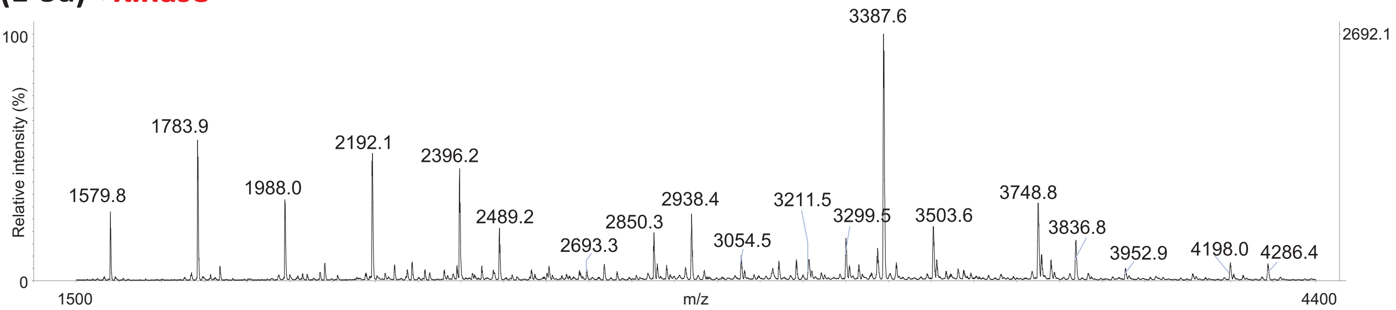

(1-1b) K88

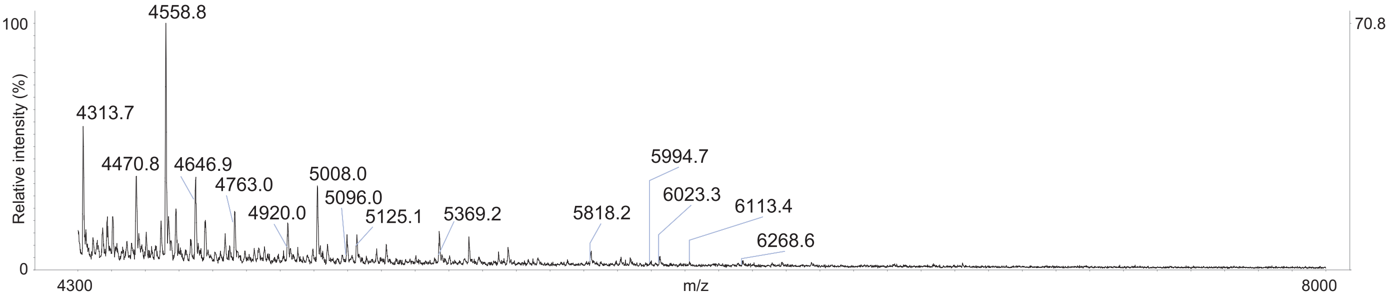

(1-2b) K20

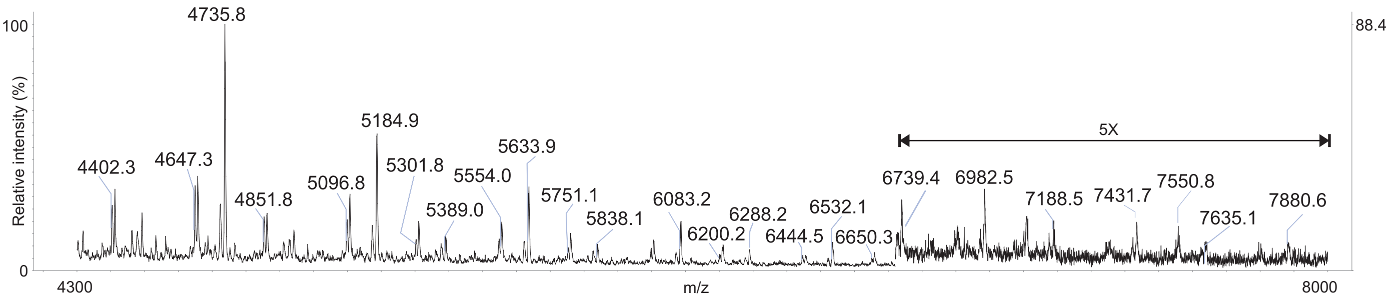

(1-3b) +GNE

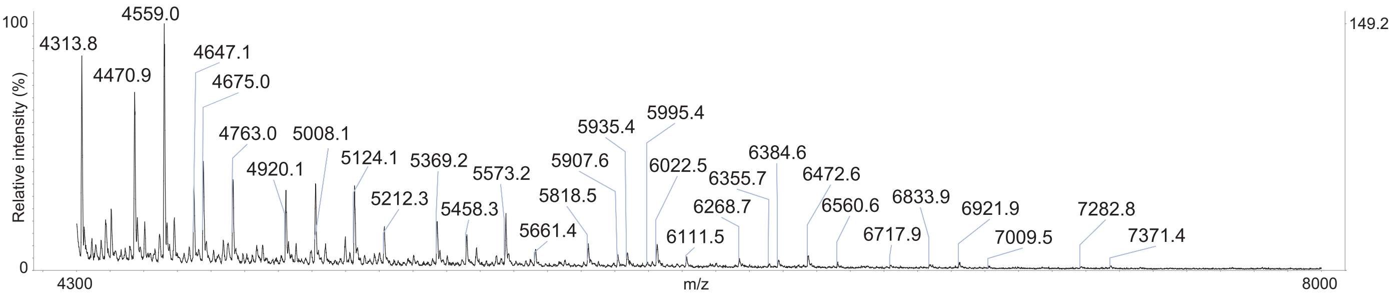

(1-4b) +D176V

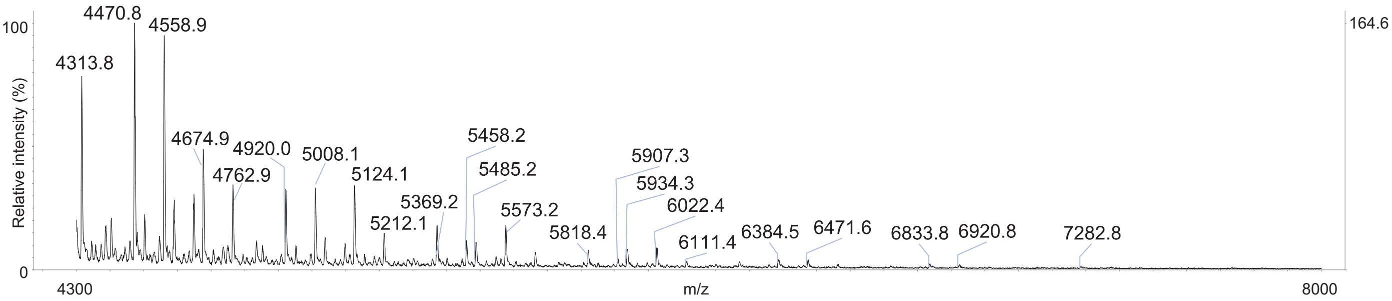

(1-5b) +Kinase

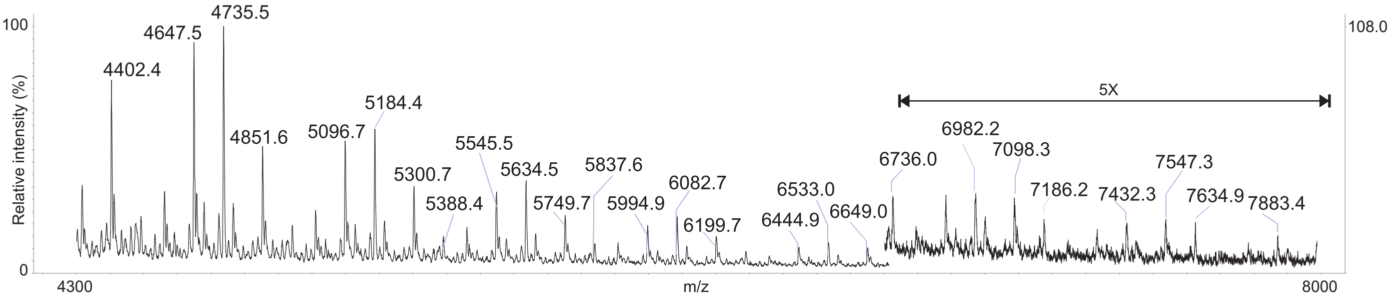

Supplementary Table 1

Tabulated MALDI-TOF MS data of permethylated N-glycans from K88, K20, +GNE, +D176V and +Kinase samples with proposed structures and relative intensities. The relative intensities are presented as percentage calculated by dividing the signal intensities at the indicated mass by the total intensities of all complex N-glycans within a sample. The intensities were extracted using the glyco-bioinformatics tool Glycoworkbench (Ceroni A., *et al.* 2008). The relative intensities values from these tables were used for generating the pie charts in Figure 6. Structural assignments are based on monosaccharide composition (obtained by MALDI-TOF MS), fragmentation analyses (MALDI-TOF/TOF MS/MS), and knowledge of glycan biosynthetic pathways. All molecular ions are $[M+Na]^+$. Sugar symbol key: galactose (yellow circle), mannose (green circle), N-acetylglucosamine (blue square), fucose (red triangle), and N-Acetylneuraminic acid (purple diamond).

Mass (m/z)	Cartoon depiction of proposed structure	Relative intensities (%)				
		K88	K20	+GNE	+D176V	+Kinase
**2070**	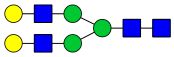	4.04	1.92	1.92	1.73	1.56
**2244**	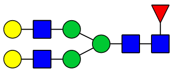	9.70	3.65	2.93	3.25	2.83
**2315**	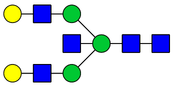	1.59	1.98	1.15	0.84	1.83
**2489**	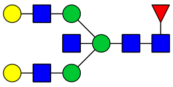	5.01	9.29	3.79	3.51	8.17
**2693**	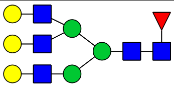	1.49	1.71	0.90	0.82	1.60
**2850**	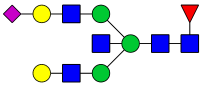	12.09	2.46	9.96	9.99	6.54
**2938**	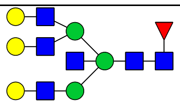	4.85	8.81	3.58	3.37	8.13
**2966**	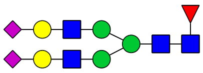	1.11	0.00	2.07	2.73	0.71
**3054**	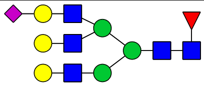	3.99	1.13	4.51	4.30	3.03
**3211**	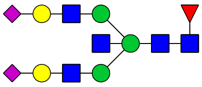	4.80	0.00	6.20	9.12	1.53
**3213**	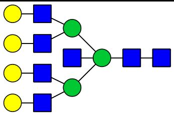	0.00	1.82	0.00	0.00	2.12
**3299**	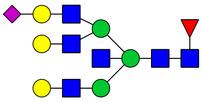	9.59	1.76	7.47	7.33	4.82
**3387**	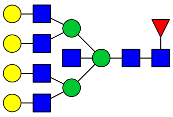	12.36	49.75	14.17	12.97	27.48
**3415**	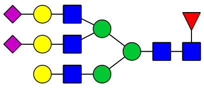	1.14	0.00	2.02	2.49	0.00

Mass (m/z)	Cartoon depiction of proposed structure	Relative intensities (%)				
		K88	K20	+GNE	+D176V	+Kinase
**3504**	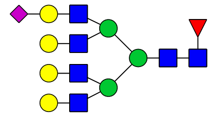	4.12	2.01	6.80	5.21	5.69
**3660**	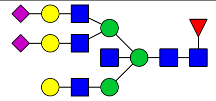	1.56	0.00	2.53	2.69	0.00
**3749**	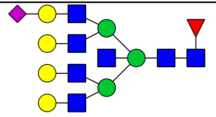	11.36	2.62	12.03	11.13	7.59
**3777**	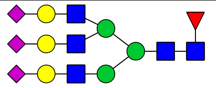	2.07	0.00	0.87	1.29	0.00
**3837**	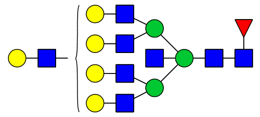	0.00	3.54	0.00	0.00	3.77
**3864**	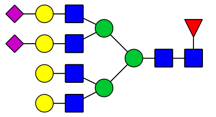	1.22	0.00	3.15	3.38	0.00
**3953**	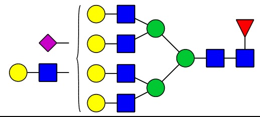	0.00	0.43	0.00	0.00	1.14
**4022**	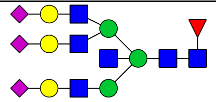	0.29	0.00	0.62	0.93	0.00
**4110**	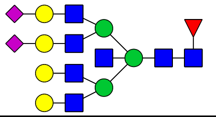	2.53	0.00	4.80	5.22	0.00
**4198**	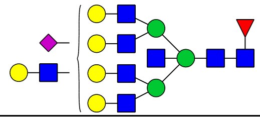	1.25	0.60	1.42	1.20	1.43
**4226**	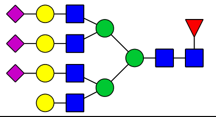	0.41	0.00	0.80	1.01	0.00
**4286**	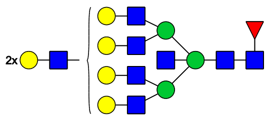	0.00	1.19	0.00	0.00	1.42
**4314**	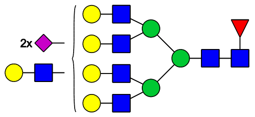	0.34	0.00	0.71	0.70	0.00
**4402**	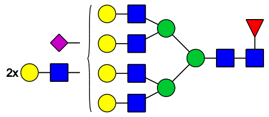	0.00	0.31	0.00	0.00	0.67
**4471**	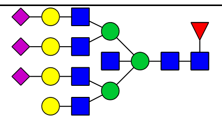	0.30	0.00	0.70	0.72	0.00

Mass (m/z)	Cartoon depiction of proposed structure	Relative intensities (%)				
		K88	K20	+GNE	+D176V	+Kinase
**4559**	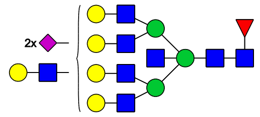	0.56	0.00	0.87	0.80	0.00
**4647**	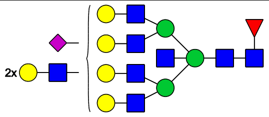	0.29	0.29	0.35	0.32	0.74
**4675**	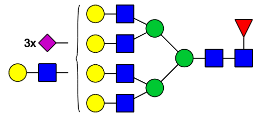	0.23	0.00	0.39	0.37	0.00
**4735**	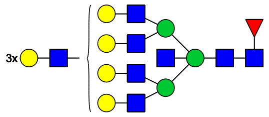	0.00	0.68	0.00	0.00	0.73
**4763**	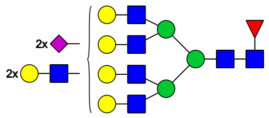	0.14	0.00	0.36	0.28	0.00
**4851**	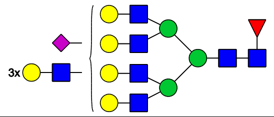	0.00	0.20	0.00	0.00	0.43
**4920**	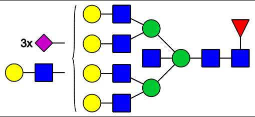	0.14	0.00	0.24	0.26	0.00
**5008**	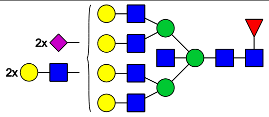	0.17	0.00	0.30	0.22	0.00
**5036**	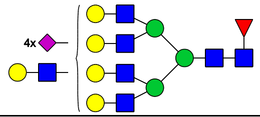	0.06	0.00	0.15	0.10	0.00
**5096**	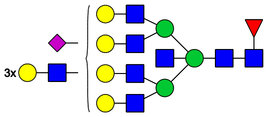	0.09	0.21	0.15	0.12	0.41
**5124**	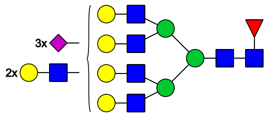	0.14	0.00	0.26	0.24	0.37
**5185**	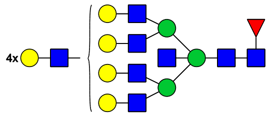	0.00	0.97	0.00	0.00	1.09
**5212**	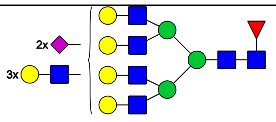	0.08	0.00	0.16	0.11	0.40
**5300**	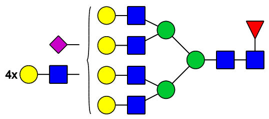	0.00	0.19	0.00	0.00	0.27

Mass (m/z)	Cartoon depiction of proposed structure	Relative intensities (%)				
		K88	K20	+GNE	+D176V	+Kinase
**5370**	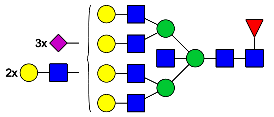	0.09	0.00	0.16	0.13	0.00
**5389**	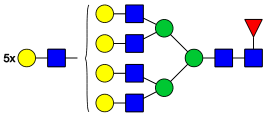	0.00	0.14	0.00	0.00	0.18
**5458**	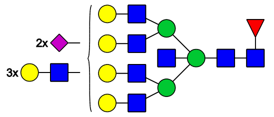	0.20	0.41	0.23	0.18	0.35
**5486**	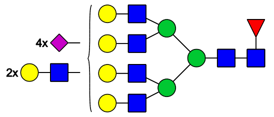	0.08	0.00	0.09	0.09	0.00
**5546**	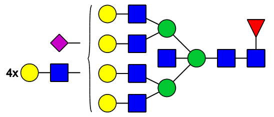	0.05	0.18	0.13	0.07	0.24
**5574**	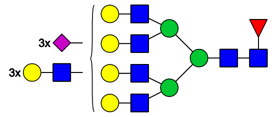	0.14	0.00	0.14	0.12	0.00
**5634**	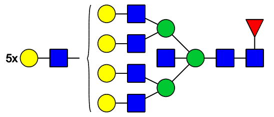	0.00	0.21	0.00	0.00	0.44
**5662**	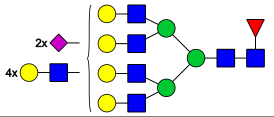	0.07	0.00	0.09	0.06	0.00
**5750**	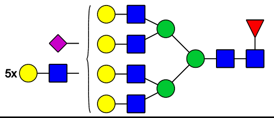	0.00	0.12	0.00	0.00	0.19
**5819**	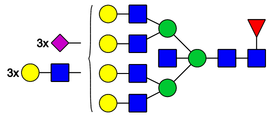	0.05	0.00	0.09	0.06	0.00
**5838**	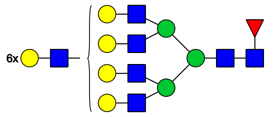	0.00	0.10	0.00	0.00	0.13
**5907**	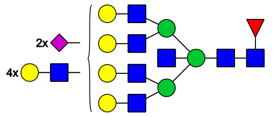	0.08	0.00	0.10	0.07	0.23
**5935**	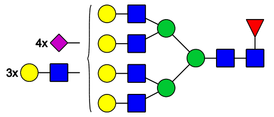	0.07	0.00	0.11	0.06	0.00
**5995**	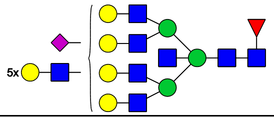	0.00	0.16	0.07	0.06	0.15

Mass (m/z)	Cartoon depiction of proposed structure	Relative intensities (%)				
		K88	K20	+GNE	+D176V	+Kinase
**6023**	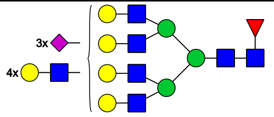	0.08	0.00	0.08	0.05	0.00
**6083**	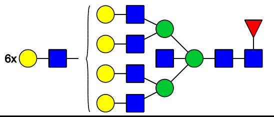	0.00	0.16	0.00	0.00	0.14
**6111**	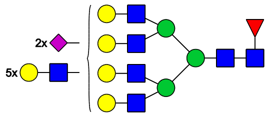	0.00	0.00	0.08	0.05	0.00
**6199**	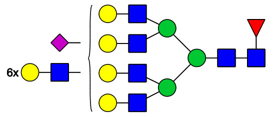	0.00	0.09	0.00	0.00	0.19
**6287**	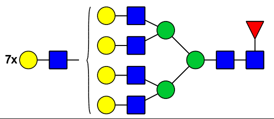	0.00	0.13	0.00	0.00	0.10
**6356**	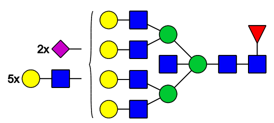	0.00	0.00	0.05	0.05	0.00
**6384**	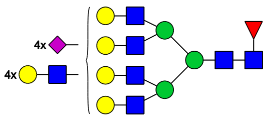	0.00	0.00	0.06	0.06	0.00
**6444**	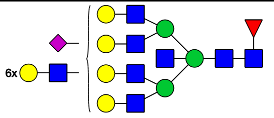	0.00	0.08	0.00	0.00	0.15
**6472**	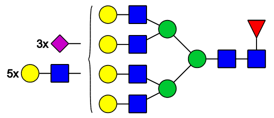	0.00	0.00	0.07	0.04	0.00
**6532**	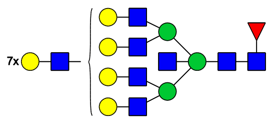	0.00	0.08	0.00	0.00	0.14
**6560**	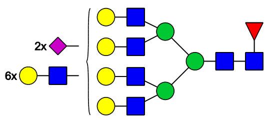	0.00	0.00	0.04	0.04	0.00
**6648**	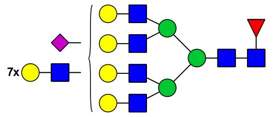	0.00	0.09	0.00	0.00	0.13
**6736**	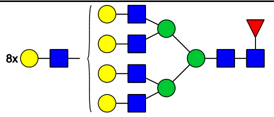	0.00	0.11	0.00	0.00	0.08
**6833**	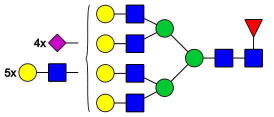	0.00	0.00	0.04	0.02	0.00

Mass (m/z)	Cartoon depiction of proposed structure	Relative intensities (%)				
		K88	K20	+GNE	+D176V	+Kinase
**6981**	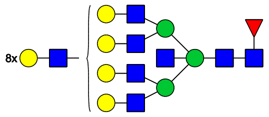	0.00	0.08	0.00	0.00	0.09
**6921**	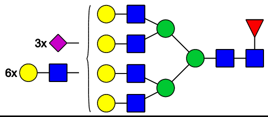	0.00	0.00	0.04	0.03	0.00
**7098**	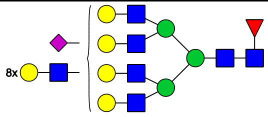	0.00	0.07	0.00	0.00	0.13
**7186**	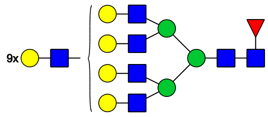	0.00	0.08	0.00	0.00	0.06
**7283**	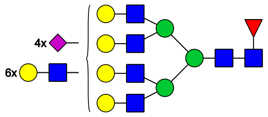	0.00	0.00	0.02	0.02	0.00
**7371**	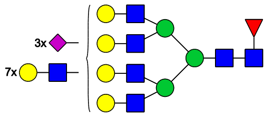	0.00	0.00	0.03	0.00	0.00
**7431**	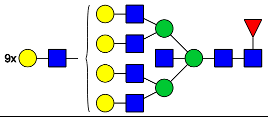	0.00	0.05	0.00	0.00	0.09
**7547**	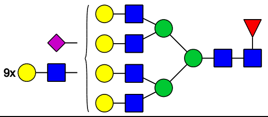	0.00	0.05	0.00	0.00	0.07
**7635**	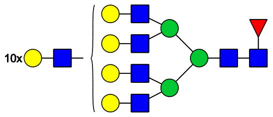	0.00	0.05	0.00	0.00	0.09
**7880**	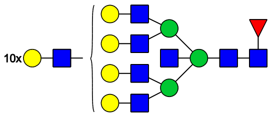	0.00	0.04	0.00	0.00	0.07
**7996**	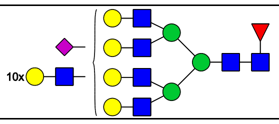	0.00	0.00	0.00	0.00	0.05
**Total**		100.00	100.00	100.00	100.00	100.00
